# Fallopian tube secreted protein affects ovarian metabolites in high grade serous ovarian cancer

**DOI:** 10.3389/fcell.2022.1042734

**Published:** 2022-11-07

**Authors:** Tova M. Bergsten, Sarah E. Levy, Katherine E. Zink, Hannah J. Lusk, Melissa R. Pergande, Stephanie M. Cologna, Joanna E. Burdette, Laura M. Sanchez

**Affiliations:** ^1^ Burdette Lab, College of Pharmacy, University of Illinois Chicago, Chicago, IL, United States; ^2^ Sanchez Lab, University of California, Santa Cruz, Department of Chemistry and Biochemistry, Santa Cruz, CA, United States; ^3^ Sanchez Lab, College of Pharmacy, University of Illinois Chicago, Chicago, IL, United States; ^4^ Cologna Lab, University of Illinois Chicago, Department of Chemistry, Chicago, IL, United States

**Keywords:** ovarian cancer, imaging mass spectrometry (IMS), fallopian tube, tumor micro environment, proteomics, SPARC

## Abstract

High grade serous ovarian cancer (HGSOC), the most lethal histotype of ovarian cancer, frequently arises from fallopian tube epithelial cells (FTE). Once transformed, tumorigenic FTE often migrate specifically to the ovary, completing the crucial primary metastatic step and allowing the formation of the ovarian tumors after which HGSOC was originally named. As only the fimbriated distal ends of the fallopian tube that reside in close proximity to the ovary develop precursor lesions such as serous tubal intraepithelial carcinomas, this suggests that the process of transformation and primary metastasis to the ovary is impacted by the local microenvironment. We hypothesize that chemical cues, including small molecules and proteins, may help stimulate the migration of tumorigenic FTE to the ovary. However, the specific mediators of this process are still poorly understood, despite a recent growth in interest in the tumor microenvironment. Our previous work utilized imaging mass spectrometry (IMS) to identify the release of norepinephrine (NE) from the ovary in co-cultures of tumorigenic FTE cells with an ovarian explant. We predicted that tumorigenic FTE cells secreted a biomolecule, not produced or produced with low expression by non-tumorigenic cells, that stimulated the ovary to release NE. As such, we utilized an IMS mass-guided bioassay, using NE release as our biological marker, and bottom-up proteomics to demonstrate that a secreted protein, SPARC, is a factor produced by tumorigenic FTE responsible for enhancing release of ovarian NE and influencing primary metastasis of HGSOC. This discovery highlights the bidirectional interplay between different types of biomolecules in the fallopian tube and ovarian microenvironment and their combined roles in primary metastasis and disease progression.

## 1 Introduction

High grade serous ovarian cancer (HGSOC) is the most lethal subtype of ovarian cancer, and can originate from fallopian tube epithelium (FTE). Transformed tumorigenic FTE located in the distal fimbriated ends of the fallopian tube can migrate to the ovary completing the crucial primary metastatic step to form tumors in the ovary (Medeiros et al., 2006; Meserve et al., 2017; Bergsten et al., 2020; Reavis and Drapkin, 2020). The chemical exchange responsible for this migration is still poorly understood, despite recent interest in understanding the tumor microenvironment (TME). To address this gap, studies have begun to utilize varied forms of mass spectrometry to investigate the role of the metabolome in the TME. For instance, UPLC-MS has been used to identify mainly lipid and peptide metabolites that can detect early HGSOC in a mouse model (Sah et al., 2022). Additionally, matrix-assisted laser desorption/ionization (MALDI) mass spectrometry has been used to map the differences in the spatial distribution of lipid metabolites between control and HGSOC samples (Jones et al., 2015). While most studies focus on large molecules like proteins and lipids, a recent meta-analysis of tumor tissues identified changes in small molecule metabolites when compared with normal tissues, suggesting small molecules may also be involved in tumor development (Reznik et al., 2018). However, these studies have not explored the metabolome in living co-culture systems, which limits their ability to study the dynamic molecular information associated with multi-organ microenvironments.

Our previous work using imaging mass spectrometry (IMS) allowed us to analyze changes in the metabolome that arose from interactions between tumorigenic fallopian tube cell models and murine ovarian explants, a site of primary metastasis. In this *in vitro* experiment, murine ovarian explants were surrounded and co-cultured with a population of murine oviductal epithelial (MOE, equivalent to human FTE) cells in a low-melting agarose matrix. Norepinephrine (NE), an adrenergic receptor agonist, was detected in significantly higher abundance from the ovarian tissue in the presence of tumorigenic MOE cells with a silenced PTEN gene (MOE PTEN^shRNA^), compared to non-tumorigenic MOE cells (MOE SCR^shRNA^ cells, encoding for a scrambled shRNA) (Zink et al., 2018). As such, IMS enabled us to confirm that small molecules, like NE and testosterone, were altered by the interaction of tumorigenic precursor tissue and organs of metastatic colonization (Zink et al., 2018; Colina et al., 2021). Of further interest is the established role of these small molecules in ovarian cancer metastasis, especially NE considering that inhibition of its signaling decreases metastasis *in vivo* (Armaiz-Pena et al., 2013).

Subsequently, our results led us to hypothesize that rather than a singular shift in a select group of small molecules, there may be a sequence of events that ultimately triggered NE release. Our previously described IMS co-culture platform presented a unique opportunity to utilize living cell culture models to explore the possible dynamic and temporal signaling events. We predicted that tumorigenic FTE cells could be secreting a preceding signal, not produced or produced with low expression by non-tumorigenic cells, that ultimately influences the ovary to release NE. We utilized our IMS platform to employ a mass-guided bioassay, with ionization and detection of NE from the ovary as our positive readout to identify factors in the conditioned media of tumorigenic FTE. Ultimately, the proteinaceous fraction of this conditioned media caused the greatest ionization of NE from the ovary. Using bottom-up proteomics, we identified a secreted protein, SPARC (secreted protein acidic and rich in cysteine), as the likely factor produced by tumorigenic FTE responsible for enhancing the release of ovarian NE and thus influencing the primary metastasis of HGSOC. An overexpression construct of SPARC resulted in increased ionization of NE from the ovary highlighting the dynamic interplay between different classes of biomolecules in the ovarian microenvironment.

## 2 Materials and methods

### 2.1 Cell culture

Murine oviductal epithelial (MOE WT, equivalent of human fallopian tube epithelial cells) cells were donated by Dr. Barbara Vanderhyden from the University of Ottawa. SKOV3 cells were purchased from ATCC (HTC-77). MOE SCR^shRNA^ and MOE PTEN^shRNA^ cells are from the Burdette Lab and have been validated (Dean et al., 2019).

MOE SCR^shRNA^ and MOE PTEN^shRNA^ cells were maintained in ɑMEM with Earle’s salts, ribonucleosides, deoxyribonucleosides, and l-glutamine (Thomas Sci 10–022-CV) supplemented (in 500 mL bottles) with 11 µL gentamicin (50 mg/mL stock, Cellgro 30–005-CR), 5 mL l-glutamine (GIBCO 25030–081), 275 µL pen/strep (10,000 U/mL or 100X stock, Thermo Fischer 15140–122), 10 µL EGF (0.1 mg/mL stock, Roche 855731), 550 µL ITS (1000X stock, Roche 1074547), 10 µL β-estradiol (1 mg/mL in 100% EtOH, Sigma Aldrich E2257), and 10% fetal bovine serum (FBS). SKOV3 cells were maintained in McCoy’s 5A (modified) medium (GIBCO 1660108) supplemented with 1.1 g sodium bicarbonate and 1% pen/strep (Thermo Fisher 15140–122), and 10% FBS. All cells were maintained in T-75 flasks, incubated at 37°C and 5% CO_2_, and passaged every 3–4 days.

### 2.2 siRNA transfection

MOE PTEN^shRNA^ cells were plated in 6 well plates at a density of 75,000 cells per well 24 h prior to transfection. A total of 400 ng/mL of SPARC endoribonuclease small interfering RNA (siRNA) (Sigma-Aldrich EMU088951) was transfected into MOE PTEN^shRNA^ cells using Mirus TransIT X2 (Mirus Bio LLC MIR6000) according to manufacturer’s protocol. Media was changed after 5 h, and cells were split and re-seeded at 48 h post-transfection into a 10 cm plate. Cells and conditioned media were then incubated for 48 h and collected for protein analysis. A plasmid that expresses mCherry was used as a positive control. A universal negative control siRNA was used as a negative control (Sigma-Aldrich SIC001).

### 2.3 Ovary dissection

CD-1 mice were obtained from in-house breeding. Animals were housed in a temperature and light (12L:12D) controlled environment. Water and food were provided *ad libitum*. All animals were treated in accordance with the National Institutes of Health Guide for the Care and Use of Laboratory Animals. On day 16–18 after birth of pups, the ovaries were removed dissected free of the uterus, fallopian tube, and bursa using a dissecting microscope (Leica MZ6).

### 2.4 Conditioned media

**FIGURE 1:**
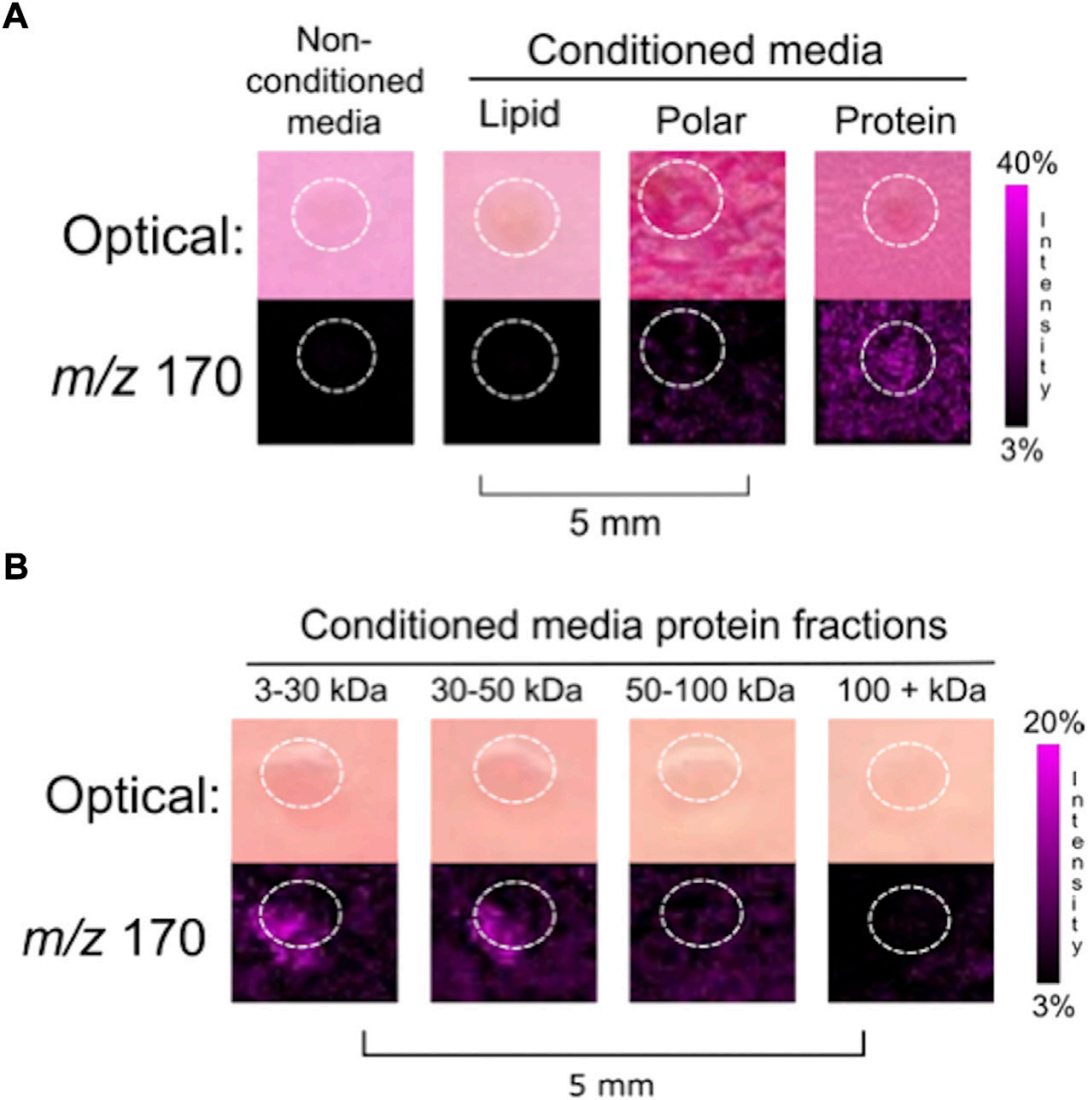
Fractionation of **(A)** MOE PTEN^shRNA^ conditioned media into biomolecular classes generated three generalized fractions: lipids, polar compounds, and proteins. Empty media was included as a negative control. The co-culture of a murine ovary with the protein fraction resulted in the release of ovarian NE. **(B)** Finer fractionation of the protein collection was done to generate four collections of limited mass ranges. Ovarian NE was released in co-culture with both the 3–30 kDa and 30–50 kDa protein fractions of MOE PTEN^shRNA^ conditioned media. Ovaries are circled with a white dotted line. IMS Method A was used for this figure.

**FIGURE 2:**
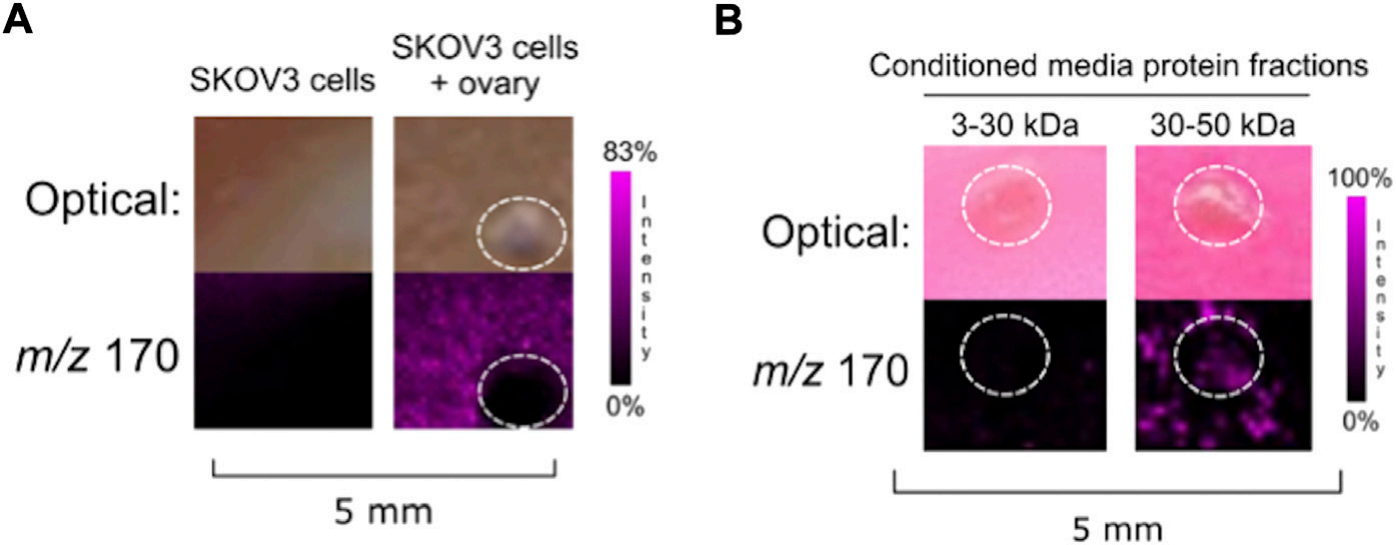
**(A)** Co-culture of SKOV3 cells with ovarian explant resulted in NE ionization. **(B)** SKOV3 conditioned media protein 30–50 kDa fraction induced ovarian NE release. Ovaries are circled with a white dotted line. IMS Method A was used for this figure.

Conditioned media for Figure 1 and Figure 2 were collected by allowing cells to incubate in normal cell culture media (see above) for 4 days at 37°C and 5% CO_2_ in T-75 flasks. Many of the subsequent analyses (Figure 4 and Figure 5) for the identification of proteins required BCA assays to determine protein concentration. Since the conventional media used for MOE cells is rich in proteins and phenol red interferes with the colorimetric readout of BCA analysis, these experiments utilized conditioned media that were serum-free and phenol-red free.

For these later analyses, MOE SCR^shRNA^ and MOE PTEN^shRNA^ cells were maintained in phenol red- and serum-free minimum essential medium, alpha (ɑMEM) with nucleosides (GIBCO 41061037) supplemented (in 500 mL bottles) with 11 µL gentamicin (50 mg/mL stock, Cellgro 30–005-CR), 5 ml l-glutamine (GIBCO 25030–081), 275 µL pen/strep (10,000 U/mL or 100X stock, Thermo Fischer 15140–122). SKOV3 cells were maintained in phenol red- and serum-free RPMI 1640 medium (GIBCO 1185055) supplemented with 1.1 g sodium bicarbonate and 1% pen/strep (Thermo Fischer 15140–122). All cells were maintained in T-75 flasks, incubated at 37°C and 5% CO_2_ for 4 days. To ensure biological consistency, IMS analyses of biomolecular fractions and protein fractions were repeated using serum-free and phenol red-free conditioned media, yielding the same results as with conventional media (data not shown).

### 2.5 Fractionation of conditioned media into biomolecular classes

Conditioned media from T-75 flasks (10 mL) were concentrated to approximately 4 mL using a centrifugal evaporator (Labconco CentriVap Concentrator (7810016) and CentriVap Cold Trap (7385020)) at 35°C to accommodate volumes of spin column filters (Millipore Sigma UFC8003). To generate the protein fraction, conditioned media was spun in a 3 kDa spin column at 4000 rpm and 25°C for 40 min in a centrifuge using a swing rotor (Eppendorf Centrifuge 5810 R). Material over 3 kDa was collected from the filter into a pre-weighed microcentrifuge tube, and the filter was rinsed and collected twice with 100 µL DI water, which was combined with the protein material. The flow through media was collected and further partitioned into lipid and polar fractions using a separatory funnel. Chloroform (3 × 1 mL) was added to the media and gently shaken. The lipid fraction was collected and the aqueous flow through was transferred to pre-weighed microcentrifuge tubes and dried *in vacuo*.

### 2.6 Fractionation of conditioned media into protein fractions

The material retained by the 3 kDa spin column was further fractioned by successive centrifugation through spin columns with molecular weight cut-off filters in the following order: 100 kDa (Millipore Sigma UFC8100), 50 kDa (Millipore Sigma UFC8050), 30 kDa (Millipore Sigma UFC8030), spun at 4000 rpm (Eppendorf Centrifuge 5810 R). This generated four fractions of molecular weight ranges: >100 kDa, 50–100 kDa, 30–50 kDa, and 3–30 kDa (Supplementary Figure S1). Each concentrate was transferred to a pre-weighed microcentrifuge tube and the filter was rinsed twice with 100 µL of DI water which was transferred to the tube. All fractions were dried *in vacuo*.

### 2.7 IMS setup of conditioned media fractions

General setup followed the protocol established in Zink et. al., except that conditioned media was used in place of a cellular mixture for co-culture with halved murine ovaries (Zink et al., 2018). Low-melting agarose (2%, Sigma A9414) was liquified from a solid at 70°C. All conditioned media fractions were normalized to the fraction with the lowest weight, generating the highest normalized concentration possible into 150 µL of media. Then, conditioned media fractions were mixed 1:1 with the liquified 2% low-melting agarose. Halved ovaries were placed in the center of wells in an 8-well chamber (Lab-Tek 177445) adhered to an ITO-coated glass slide (Bruker 8237001). Each condition (300 µL) was plated into wells, avoiding air bubbles, and ensuring the ovarian tissue remained in the center of the well. In conditions with addition of exogenous SPARC, the compound was resuspended in DI water and used at 50 ng/150 µL (*Mus musculus*, R&D Systems 942SP050). Slides were placed in the humidified incubator at 37°C and 5% CO_2_ and left to incubate for 4 days. After 4 days, the agarose plugs were cut away from the 8-well chamber and the chamber was detached from the slide. The agarose plugs were dried on the ITO-coated glass slide in an oven at 37°C for 2–4 h, monitored closely for wrinkling of the agarose (Lusk et al., 2022).

### 2.8 Matrix application

Matrix application protocol was used as previously described, aside from an increase to the concentration to 10 mg/ml (Zink et al., 2018).

### 2.9 MALDI IMS analysis

Due to differing access to instrumentation, data were collected in two different ways. In each figure legend, the method is noted to indicate how the IMS data were generated. All RAW and processed data are available at the MassIVE (massive.ucsd.edu) accession number MSV000089866.

Method A: Prior to IMS analysis, slides were scanned at 1200 dpi, and resulting images were used to guide irradiation. IMS data were acquired using flexControl v 3.4 at 50 μm spatial resolution on an Autoflex Speed LRF instrument (Bruker Daltonics) over the mass range 100–2000 Da. In positive reflectron mode, laser power was set to 40%, laser width to 2 (small), and reflector gain to 2.0 ×. For each raster point, 500 laser shots at 2000 Hz were shot in a random walk method. Data were subsequently analyzed in flexImaging v 4.1 × 64 (Bruker Daltonics). All spectra were normalized to the total ion count (TIC). The instrument was calibrated manually using phosphorus red.

Method B: Prior to IMS analysis, slides were scanned using Tissue Scout (Bruker Daltonics), and resulting images were used to guide irradiation. IMS data were acquired using timsControl v2.0.51.0_9669_1571 and flexImaging 5.1 software at 100 μm spatial resolution on a timsTOF fleX instrument (Bruker Daltonics). The data were collected using the mass range 50–1500 Da in positive mode with laser power set to 90.006%, and laser width to 100 µm imaging. For each raster point, 1,000 laser shots at 1,000 Hz were shot. Data were subsequently analyzed in SCiLS™ Lab version 2021b core (Bruker Daltonics). The instrument was calibrated manually using phosphorus red.

### 2.10 Digestion of 3–50 kDa proteins for Q-Exactive LC-MS/MS

Protein concentrations for concentrated conditioned media samples were determined *via* BCA assay and 50 µg of protein was enzymatically digested prior to mass spectrometry analysis. Ammonium bicarbonate (ABC, 50 mM in DI H_2_O) was added to create a uniform sample volume. Dithiothreitol (DTT) was added to the digest at final concentration of 10 mM and incubated for 15 min in a 55°C water bath. After cooling to room temperature, iodoacetamide was added to a final concentration of 30 mM and then incubated for 20 min in a 37°C water bath. Trypsin was added (1 ng trypsin/20 ng protein) and samples incubated overnight in a 37°C water bath. After overnight incubation, the resulting tryptic peptides were enriched and desalted *via* C18 ZipTips using the manufacturer’s suggested protocol and eluant dried *in vacuo*. Solution was resuspended in 0.1% formic acid to 1 μg/μL prior to LC-MS/MS analysis.

### 2.11 LC-MS/MS proteomics analysis

The peptide digests were analyzed as described previously by Pergande et al. Using a Q Exactive Hybrid Quadrupole-Orbitrap mass spectrometer (Thermo Scientific) which was interfaced to an Agilent 1260 nano/capillary high-performance liquid chromatography (LC) system (Pergande et al., 2021). Mobile phase A was 0.1% formic acid in water and mobile phase B was 0.1% formic acid in acetonitrile. First, peptides were loaded onto an Zorbax 300SB-C18 trap cartridge (Agilent Technologies) and washed with 0.1% formic acid at a flow rate of 2 μL/min and 10 min. Next, the peptides were chromatographically resolved on a Zorbax 300SB-C18 column (3.5 µm i.d. × 150 mm, particle size 5µm, pore size 100Å, Agilent Technologies) using a 5–60% B 60-min linear gradient for at a flow rate of 250 nL/min. The mass spectrometer was operated in data-dependent acquisition mode. The instrument conditions were set to acquire data by data-dependent parameters, utilizing the top 10 peptides per duty cycle, switching between MS and MS/MS. The scan mode for data acquisition in MS1 was set to perform full scan (*m/z* 375–1600) at a resolution of 70,000 using automatic gain control and with a target of 1 × 10^6^ ions. For MS/MS data acquisition the resolution was set to 17,500 with a target of 1 × 10^5^ ions. The isolation window set was *m/z* 1.5, utilizing a collision energy of 27.0 eV and dynamic exclusion at 20.0 s. The source ionization had a spray voltage of 1.9 kV with a capillary temperature set to 280°C, s-lens RF, 50.0. MOE PTEN^shRNA^ and MOE SCR^shRNA^ samples (N = 5) were analyzed in duplicate (n = 2). SKOV3 samples (N = 5) were analyzed in triplicate (n = 3). All raw and mzML mass spectrometry data is publicly available at MassIVE (massive.ucsd.edu) under the accession number MSV000089866. Protein ID’s were filtered with the following parameters: abundance ratio (MOE PTEN^shRNA^/MOE SCR^shRNA^) > 1.0, molecular weight (MW) 25–40 kDa as we suspected the protein of interest was approximately 30 kDa in size, and sequence coverage >8%. For the MOE PTEN^shRNA^ and MOE SCR^shRNA^ conditioned media, we filtered for the *Mus musculus* species; for SKOV3 conditioned media, we filtered for *Homo sapiens* species.

### 2.12 LC-MS/MS data analysis

Raw data files from LC-MS/MS analysis of the trypsin digested samples were imported into Proteome Discoverer (version 2.2, Thermo Fisher) and analyzed using a label-free, relative quantitation method. Protein identifications for MOE PTEN^shRNA^ and MOE SCR^shRNA^ cohorts were obtained by searching against both the *Mus musculus* (25230 sequences) and contaminants (298 sequences) databases. Similarly, protein identifications for SKOV3 samples were obtained by searching against the *Homo sapiens* (42368 sequences) and contaminants database employing a 1% false discovery rate and the Perculator algorithm. Here, trypsin was set as the protease with two missed cleavages and searches were performed with precursor and fragment mass error tolerances set to 10 ppm and 0.02 Da, respectively, where only peptides precursors of +2, +3 and +4 were considered. Peptide variable modifications allowed during the search were oxidation (M) and deamination (NQ), whereas carbamidomethyl (C) was set as fixed modifications. Then, a label-free relative quantitation analysis was performed for MOE PTEN^shRNA^ relative to MOE SCR^shRNA^ significance determined by applying an unpaired *t*-test (*p* ≤ 0.05).

## 3 Results

### 3.1 A mass-guided approach to identify the tumorigenic FTE secreted factor driving ovarian NE production

We hypothesized that a secreted factor was produced from tumorigenic FTE and that it was responsible for inducing the release of ovarian NE. The induction of ovarian NE release occurred when ovaries were cultured with tumorigenic MOE PTEN^shRNA^ cells but not with non-tumorigenic MOE SCR^shRNA^ cells. The only engineered difference between the MOE PTEN^shRNA^ and MOE SCR^shRNA^ models is the shRNA directed against the tumor suppressor gene PTEN, which enables the MOE PTEN^shRNA^ line to form rapid cancer from intraperitoneal injection in murine models. As such, we continued to use the MOE SCR^shRNA^ as a non-tumorigenic control while investigating the tumorigenic MOE PTEN^shRNA^, its conditioned media, and potential secreted factors.

As shown in our previously described IMS co-culture model, the typical distance between the cells and the ovary in the divided chambers was 1–2 mm and the matrix supporting the culture was comprised of agarose (Zink et al., 2018). Therefore, we predicted that the FTE factor responsible is likely both secreted and readily diffusible in order to reach and interact with the ovary. To test whether secreted factors from the cells were sufficient to induce ovarian NE release, we opted to alter our co-culture model to utilize conditioned media rather than cells themselves. Woznica et al. described an innovative bioassay-guided fractionation scheme to determine the protein responsible for inducing mating in a choanoflagellate species (Woznica et al., 2017). Inspired by this approach, we aimed to fractionate conditioned media from MOE PTEN^shRNA^ cells to determine whether a specific class of biomolecule was responsible for inducing the release of NE. Supplemental Figure S1 outlines the steps taken to separate cell-free conditioned media into three biomolecular classes: lipids, polar small molecules, and proteins.

After 4 days of incubation with half of a murine ovary, all the cell free conditioned media fractions cultured with an ovary were analyzed using MALDI-TOF IMS. NE was detected from the ovary that was cultured with the protein fraction (>3 kDa) (N = 3) (Figure 1A), indicating that the FTE factor influencing ovarian NE release was proteinaceous. Further fractionation was then applied to the protein fraction to generate four protein fractions with unique mass ranges: 3–30 kDa, 30–50 kDa, 50–100 kDa, and >100 kDa. Co-incubation of ovarian tissue with these protein fractions, normalized by dry weight, resulted in the ionization of NE from both the 3–30 and 30–50 kDa protein fractions (N = 3) (Figure 1B).

### 3.2 Bottom-up proteomics yielded candidates for FTE factor

The release of ovarian NE was detected when incubated with MOE PTEN^shRNA^ secreted proteins fractionated to 3–30 kDa but not with those from MOE SCR^shRNA^ (Supplemental Figure S2). We used the mass range 3–50 kDa because the NE signal was seen in both the 3–30 kDa and 30–50 kDa co-cultures. Additionally, SDS-PAGE of the serum-free MOE PTEN^shRNA^ protein fraction in the 3–50 kDa size range generated a prominent band just below 35 kDa, which was not present in the same protein fraction from MOE SCR^shRNA^ as identified by Coomassie Blue and Zinc staining (Supplemental Figure S3). To identify the protein factor, the area surrounding this band from both MOE PTEN^shRNA^ and MOE SCR^shRNA^ fractions were digested for bottom-up proteomics. With MOE SCR^shRNA^ used as the control, digested peptides (N = 5, n = 2) were analyzed using LC-MS/MS. All identifications across runs were tallied, duplicates removed, and proteins were compared between MOE PTEN^shRNA^ and MOE SCR^shRNA^. The identification workflows for all biological replicates cumulatively identified 45 proteins in MOE PTEN^shRNA^ and 48 in MOE SCR^shRNA^. Of these proteins, 27 were shared between the cell lines, 21 were unique to MOE SCR^shRNA^, and 18 were unique to MOE PTEN^shRNA^ (Supplemental Table S1). The relative protein abundances from each cohort were used to generate a list of proteins which was filtered to select for increased fold change in the MOE PTEN^shRNA^ samples. Ultimately, three proteins were observed to have an increased abundance in the MOE PTEN^shRNA^ samples relative to the MOE SCR^shRNA^ samples. SPARC (secreted protein acidic and rich in cysteine), also known as osteonectin, had the highest sequence coverage and number of unique peptides (Table 1).

**TABLE 1:** Protein candidates filtered from semi-quantitative comparison of proteins abundant in MOE PTEN^shRNA^ when compared with MOE SCR^shRNA^. Protein IDs were filtered with the following parameters: abundance ratio (MOE PTEN^shRNA^/MOE SCR^shRNA^) > 1.0, molecular weight (MW) 25–40 kDa, Species: *Mus musculus*, and sequence coverage >8%. PSMs is peptide spectrum matches.

Description	Coverage [%]	# Peptides	# PSMs	# Unique peptides	MW [kDa]	Abundance ratio: (Sample/Control)
SPARC	31	7	36	7	34.4	1.661
Osteopontin	21	5	72	5	32.4	1.801
Glyceraldehyde-3-phosphate dehydrogenase	10	2	10	2	35.8	2.394

### 3.3 Putative protein identification in SKOV3 human ovarian cancer cells

To explore possible cross species confirmation, we investigated whether a human ovarian cancer cell line previously reported to display enhanced migration and peritoneal spread in response to NE was also capable of inducing NE production from the ovary. The ability of SKOV3 to respond to NE signaling is well-established (Sood et al., 2006; Sood et al., 2010). For instance, invasion assays indicated that SKOV3 cells increased invasion in response to NE at least 2-fold over vehicle control, and blockage of NE signaling with propranolol decreased tumor spread *in vivo* (Sood et al., 2006; Zink et al., 2018). From this data suggesting that SKOV3 cells are responsive to NE, we hypothesized that the ovary may also release NE in the presence of SKOV3 cells. Increased ovarian NE release when cultured with SKOV3 conditioned media would confirm the presence of a conserved secreted protein and provide an orthogonal cross-species cell model for validating the IMS analysis and prioritizing the bottom-up proteomics.

The IMS mass-guided assay was repeated with SKOV3 cells. When co-cultured with SKOV3 cells, ovarian NE release was detected (Figure 2A). We subsequently collected SKOV3 conditioned media and fractionated it to isolate the 3–30 and 30–50 kDa protein fractions. Ovaries incubated with the SKOV3 conditioned media protein fractions indicated that a factor secreted from the human ovarian cancer cell line in the 3–50 kDa range was also capable of inducing ovarian NE release (Figure 2B). These data suggest that both a murine fallopian tube cell line and a human ovarian cancer cell line produce a secreted factor that causes the ovary to release NE.

**FIGURE 3:**
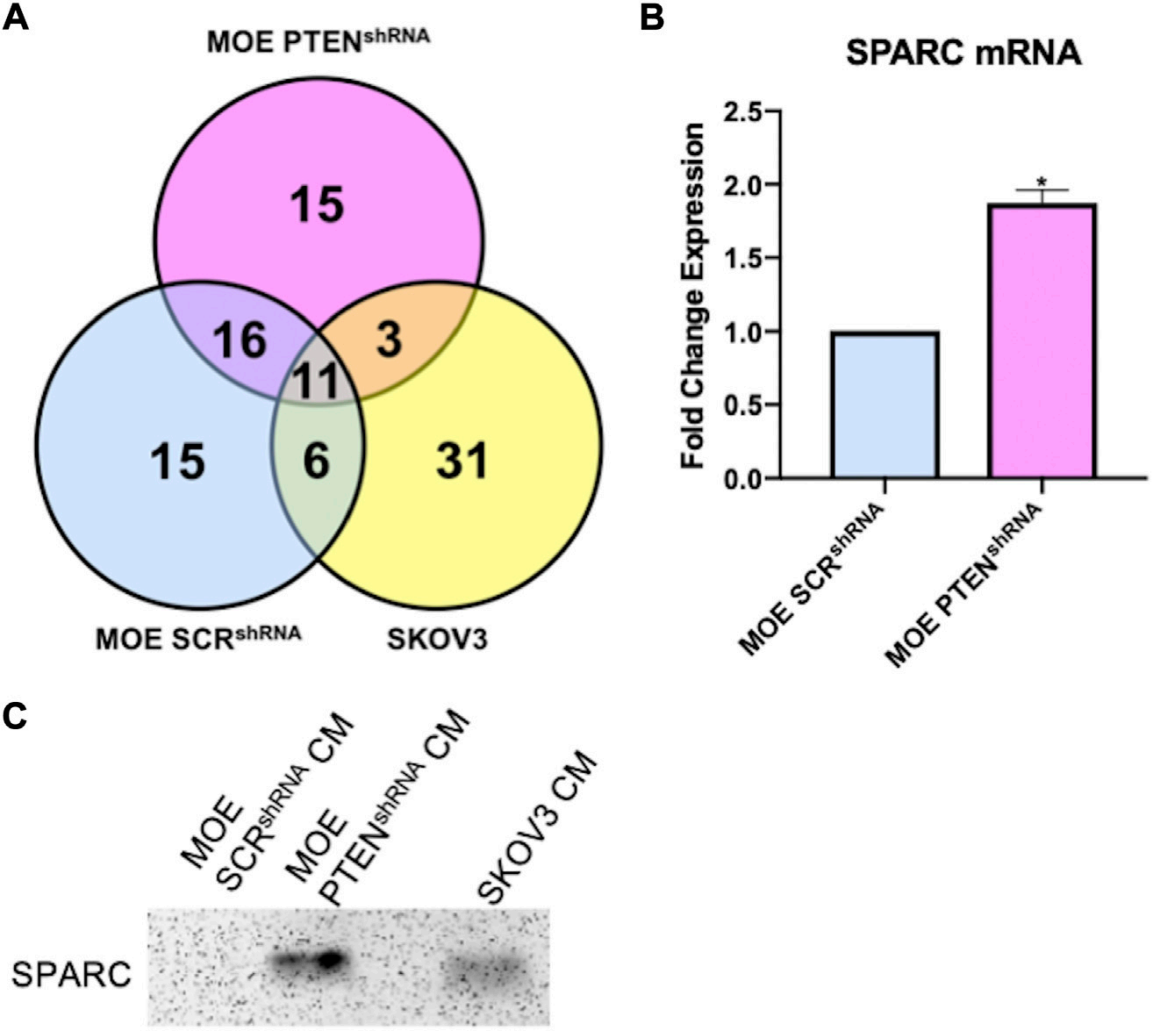
**(A)** Venn diagram of proteins identified from proteomics analysis in conditioned media 3–50 kDa samples from MOE SCR^shRNA^, MOE PTEN^shRNA^, and SKOV3. **(B)** RNA sequencing data demonstrates higher mRNA levels of SPARC in MOE PTEN^shRNA^ cells than in MOE SCR^shRNA^ cells; unpaired *t*-test, * = *p* < 0.05. **(C)** Western blot shows higher expression of SPARC protein in MOE PTEN^shRNA^ and SKOV3 conditioned media than in MOE SCR^shRNA^ conditioned media. CM stands for conditioned media.

Since our IMS assay validated that a human ovarian cancer cell line also secreted a protein that results in the production and ionization of ovarian NE in the 30–50 kDa fraction, the SKOV3 protein fractions were prepared for bottom-up proteomics analysis as described above. While the evaluation and identification of proteins in the MOE PTEN^shRNA^ fractions were assessed for relative quantitation against peptide abundances in the MOE SCR^shRNA^ collection, in the case of SKOV3, there is no equivalent engineered cell line to represent non-cancerous cells. As such, the identification of all proteins in this fraction was determined and then compared against the MOE PTEN^shRNA^ proteins. SKOV3 samples were also analyzed to identify proteins in the 3–50 kDa fraction for consistency. 51 proteins were identified from all SKOV3 samples (N = 5, n = 3), 14 of which were also identified in MOE PTEN^shRNA^, and their comparison to MOE PTEN^shRNA^ protein identifications are presented in Supplemental Table S2. Of the 14 proteins in common between SKOV3 and MOE PTEN^shRNA^, only 5 fit the filter parameters, of which SPARC had the highest number of unique peptides and second highest sequence coverage (Table 2). When we compared all the proteomic data, only three proteins were shared between SKOV3 and MOE PTEN^shRNA^ that were not shared with MOE SCR^shRNA^: galectin-1, hemopexin, and SPARC (Figure 3A). As SPARC was the only one of these 3 proteins that was within the filter parameters, SPARC was prioritized as the leading candidate protein of interest. We then confirmed with RNA-sequencing that MOE PTEN^shRNA^ cells had higher levels of SPARC expression than MOE SCR^shRNA^ (Figure 3B), and that both MOE PTEN^shRNA^ and SKOV3 conditioned media contained more SPARC than MOE SCR^shRNA^ *via* western blot (Figure 3C).

**TABLE 2:** Protein candidates from both SKOV3 and MOE PTEN^shRNA^ proteins 3–50 kDa that fulfill parameters to be FTE factor. Protein candidates from both SKOV3 and MOE PTEN^shRNA^ proteins 3–50 kDa that fulfill parameters to be FTE factor: in both *Homo sapiens* and *Mus musculus*, molecular weight (MW) 25 kDa–40 kDa, and sequence coverage >8%. PSMs is peptide spectrum matches.

Description	Coverage [%]	# Peptides	# PSMs	# Unique peptides	MW [kDa]
Insulin-like growth factor-binding protein	32	6	64	6	29
SPARC	31	7	36	7	34.4
Osteopontin	21	5	72	5	32.4
CCN family member	20	6	75	6	37.8
Glyceraldehyde-3-phosphate dehydrogenase	10	2	10	2	35.8

### 3.4 SPARC regulation of ovarian NE release

**FIGURE 4:**
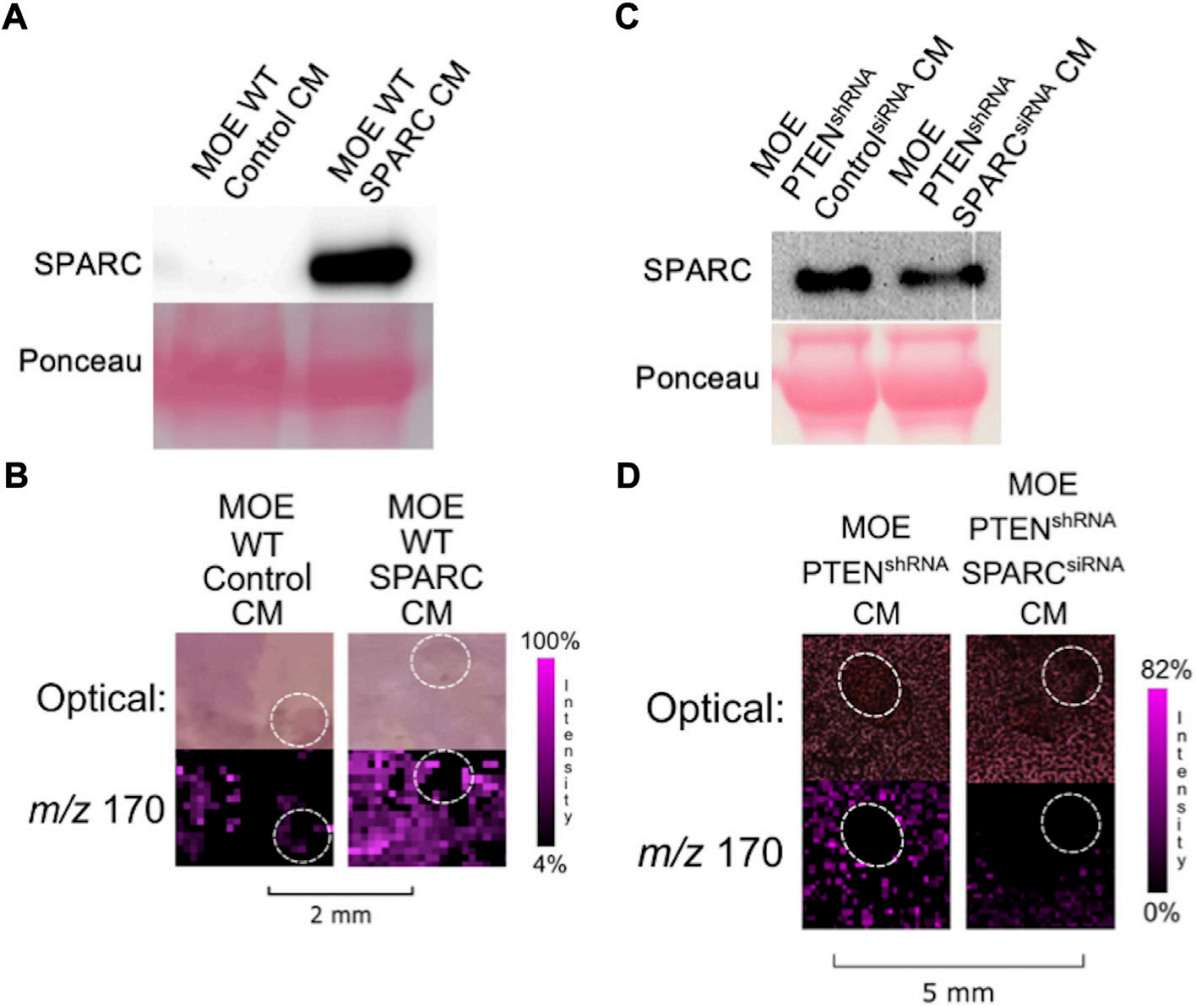
**(A)** Transfection of SPARC in MOE WT results in overexpression of SPARC protein compared to transfection with a control plasmid. **(B)** Conditioned media from MOE WT SPARC co-cultured with ovarian explant results in increased NE signal from ovary. **(C)** Transfection of SPARC^siRNA^ in MOE PTEN^shRNA^ results in knockdown of SPARC expression and less secretion of SPARC protein into conditioned media. **(D)** Conditioned media from MOE PTEN^shRNA^ SPARC^siRNA^ co-cultured with ovarian explant results in decreased NE signal from ovary. Ovaries are circled with a white dotted line. IMS Method B was used for this figure. CM stands for conditioned media.

To determine if SPARC was involved in the induction of ovarian NE release, we sought to increase endogenous expression of SPARC in MOE WT cells whose conditioned media was not previously able to induce ovarian NE release. Therefore, we transfected a SPARC overexpression plasmid in MOE WT cells and confirmed expression *via* western blot (Figure 4A). The resulting IMS images demonstrated that increasing SPARC expression in MOE WT cell conditioned media enabled the induction of ovarian NE release (Figure 4B). We also sought to determine whether it was possible for MOE PTEN^shRNA^ to maintain induction of ovarian NE release with SPARC depleted. We transfected MOE PTEN^shRNA^ cells with SPARC siRNA or control siRNA and collected conditioned media from these cells. Western blotting confirmed the knockdown of the SPARC protein had occurred in the conditioned media (Figure 4C). Next, conditioned media from these transfected cells were co-cultured with murine ovaries, and IMS was performed on these co-cultures. Analysis demonstrated that the MOE PTEN^shRNA^ SPARC^siRNA^ conditioned media induced a less intense ovarian NE signal than the conditioned media from MOE PTEN^shRNA^ control^siRNA^ (Figure 4D). These data suggest that SPARC is necessary to induce ovarian NE release.

**FIGURE 5:**
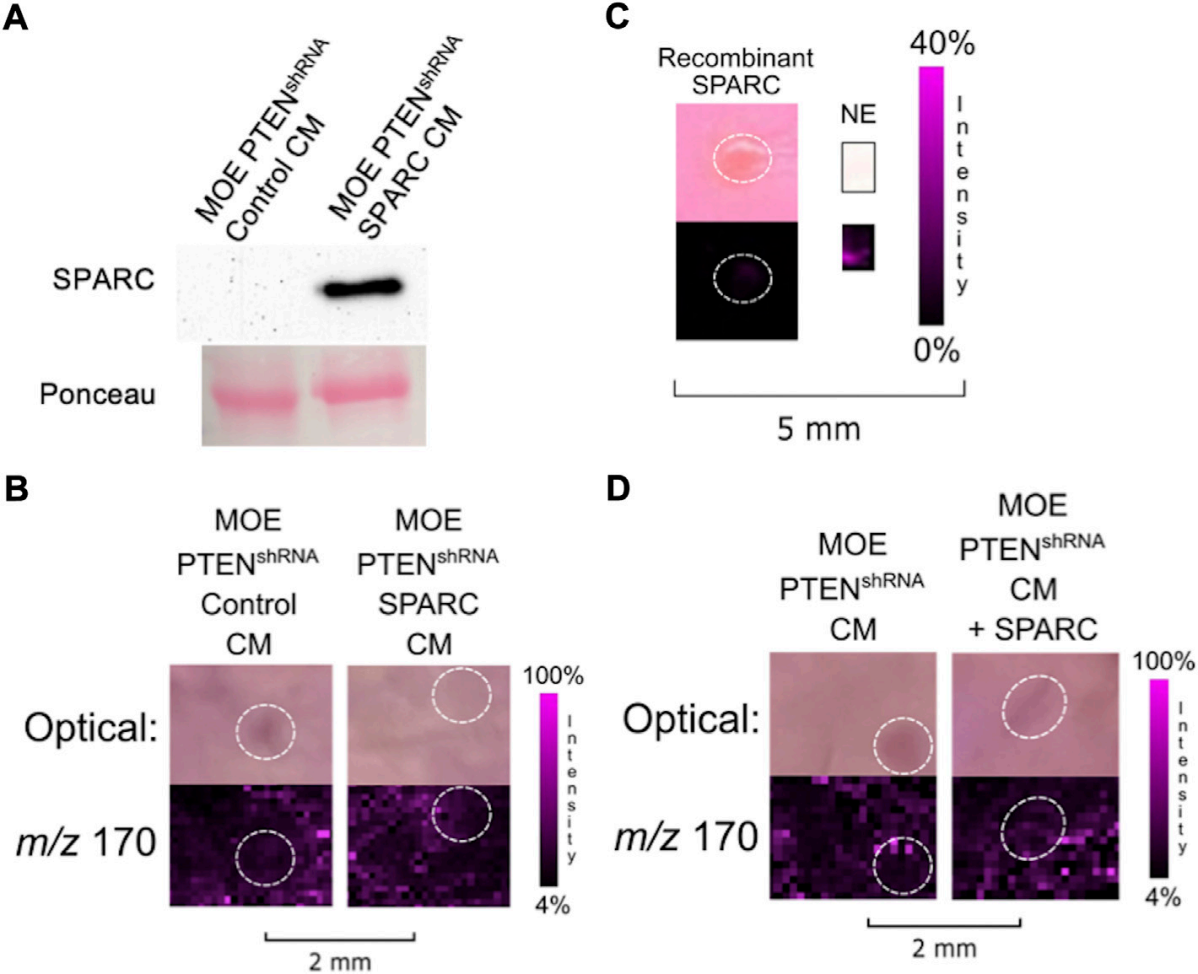
**(A)** Transfection of SPARC in MOE PTEN^shRNA^ results in overexpression of SPARC protein compared to transfection with a control plasmid. **(B)** Conditioned media from MOE PTEN^shRNA^ SPARC co-cultured with ovarian explant does not increase NE signal from ovary. **(C)** Treatment of ovary with recombinant SPARC (10 μ g/150 μ L) does not increase NE signal from ovary. **(D)** Ovarian explant treated with conditioned media from MOE PTEN^shRNA^ combined with recombinant SPARC (50 ng/150 μ L) does not increase NE signal from ovary. Ovaries are circled with a white dotted line. IMS Method B was used for this figure. CM stands for conditioned media.

We next examined whether overexpression of SPARC in MOE PTEN^shRNA^ cells would stimulate more release of NE from the ovary than previously visualized. However, while transfecting MOE PTEN^shRNA^ with the same SPARC construct increased the amount of SPARC in the resultant conditioned media (Figure 5A), it was unable to significantly alter the NE release from an ovarian explant in co-culture compared with control transfected MOE PTEN^shRNA^ conditioned media (Figure 5B). To see if SPARC alone could induce the release of ovarian NE, we treated a murine ovary with recombinant SPARC. The addition of recombinant SPARC (10 μ g/150 μ L) did not result in the release of ovarian NE as seen with IMS (Figure 5C). Additionally, when MOE PTEN^shRNA^ conditioned media is combined with recombinant SPARC (50 ng/150 μ L), the additional SPARC is not seen to increase ovarian NE release compared to MOE PTEN^shRNA^ conditioned media (Figure 5D). Hence, we posit that the SPARC secreted into conditioned media of MOE PTEN^shRNA^ cells expressed a key post-translational modification that was missing on the recombinant protein or that SPARC only elicits NE induction when combined with another factor missing in the recombinant only condition.

## 4 Discussion

High grade serous ovarian cancer, the most common and most lethal gynecologic malignancy, can originate from fallopian tube epithelium (Marquez et al., 2005; Kindelberger et al., 2007; Lee et al., 2007; Kurman, 2013; Bowtell et al., 2015; Falconer et al., 2015; Madsen et al., 2015; Perets and Drapkin, 2016; Ducie et al., 2017; Karnezis et al., 2017). Literature suggests that the primary metastasis of malignant FTE cells from the fallopian tube to the ovary is critical for the development of advanced disease. These studies are supported by clinical data which suggest that bilateral salpingectomies or salpingo-ophrectomies are protective against the development of ovarian cancer (Falconer et al., 2015; Madsen et al., 2015). However, diagnosing the disease early when it is confined to the fallopian tube is difficult given the lack of symptoms at early stages and lack of screening modalities. As recently as 2021, the United Kingdom Collaborative Trial of Ovarian Cancer Screening (UKTOCS), a massive multi-site evaluation of multiple screening mechanisms, was unable to identify a screening method that could be recommended to the general population (Menon et al., 2021). As such, a majority of women present in very late stages of disease (Howlader NN et al., 2014). Therefore, identifying the signals that are involved in this primary metastasis could improve early detection, increase opportunities for prevention, and provide new avenues for therapeutic interventions.

Our previous work innovatively applied IMS to a co-culture of tumorigenic FTE cells and ovarian explants to identify that the ovary releases NE in response to the presence of the tumorigenic cells (Zink et al., 2018; Zink et al., 2019). Since NE has been shown to increase invasiveness and worsen tumor burden in ovarian cancer models, a preceding signal that induces its release from the ovary may contribute to primary metastasis and early disease development. Therefore, we set out to determine if there was another identifiable signal at play responsible for the ovarian release of NE during early disease development.

We discovered that tumorigenic FTE cells secreted a protein in the 3–50 kDa range that was able to induce ovarian NE release. Initially, we hypothesized that the resultant ionization of NE from both the 3–30 and 30–50 kDa fractions was due to the size of the responsible protein being around 30 kDa since the pore sizes in the spin filters are not absolute cut-offs and some carry over is expected. SPARC is approximately 35 kDa in size and is therefore on the edge of the cut-off size for the spin filters. However, induction resulting from both size fractions could also be due to two separate proteins, one in the 3–30 kDa and one in the 30–50 kDa fractions. This combined action may contribute to the NE ionization signal appearing brighter and more pervasive in the total protein fraction than it does in either the 3–30 kDa or 30–50 kDa fraction images. Therefore, bottom-up proteomics on the wider 3–50 kDa fraction was conducted to ensure we were including all proteins in both ranges which may have been secreted with identifiably different abundances.

The only modulated difference between MOE PTEN^shRNA^, which induce ovarian NE ionization, and MOE SCR^shRNA^, which do not, is the shRNA that diminishes PTEN expression (Zink et al., 2018). As such, it is likely that the expression of the protein responsible for NE induction was increased due to the loss of PTEN expression. Intriguingly, SPARC production appears to be modulated by PTEN and AKT. Previous literature has demonstrated that SPARC induced effects are supported by AKT activation and attenuated by PTEN activation, presumably since PTEN activation suppresses AKT signaling (Thomas et al., 2010; Alam et al., 2013). RNA-sequencing data supports that MOE PTEN^shRNA^ cells, which have a knockdown of PTEN resulting in unchecked AKT signaling, have higher expression of SPARC than their PTEN-intact counterpart MOE SCR^shRNA^. Therefore, based on our RNA and protein expression data, FTE with a loss of PTEN and subsequent upregulation of AKT signaling have higher levels of SPARC and present the opportunity for SPARC induced NE release from the ovary.

While exogenous recombinant SPARC alone was not able to induce ovarian NE release, we were able to detect both a decrease in ovarian NE release when SPARC was knocked down in MOE PTEN^shRNA^ as well as enhanced ovarian NE release when SPARC was overexpressed in MOE WT. These data suggest that while SPARC is playing a role in ovarian NE release, recombinant SPARC is not sufficient to initiate this release alone. This lack of induction could imply that SPARC requires specific post-translational modifications that are generated in the MOE cells when it produces endogenous protein and that these modifications are not found on the recombinant protein. Alternatively, as previously mentioned, the induction of ovarian NE release may require SPARC working in tandem with another protein in the 3–50 kDa range. Additionally, neither the addition of exogenous recombinant SPARC nor the overexpression of SPARC in MOE PTEN^shRNA^ conditioned media increased ovarian NE compared to unaltered MOE PTEN^shRNA^ conditioned media. These data suggest that while MOE PTEN^shRNA^ may already produce the amount of SPARC needed to reach the threshold for ovarian NE release, higher levels of another cooperating protein may be required to further increase NE release.

**FIGURE 6:**
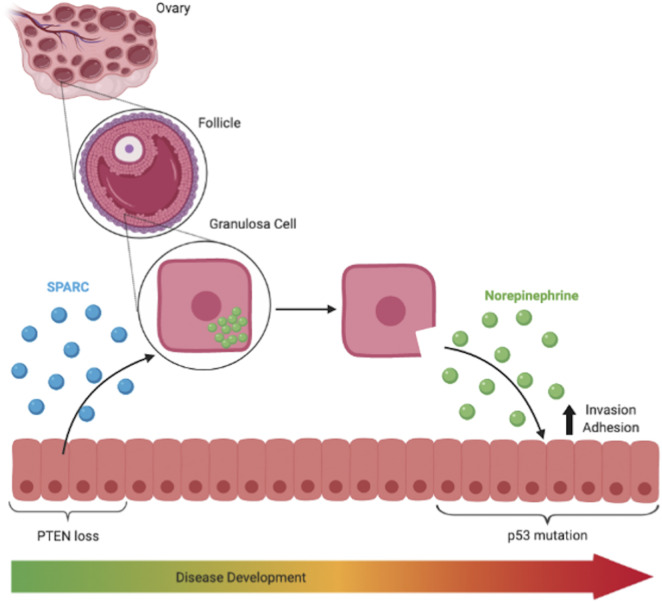
SPARC is known to upregulate production of enzymes that remodel the basement membrane in follicles and is responsible for the release of ovarian NE into the surrounding environment. NE treatment increased invasiveness and adhesive capabilities of p53 mutated cells.

SPARC is known to upregulate the production of several enzymes, namely collagenase, in the ovary that reassemble the membrane surrounding the follicle (Tremble et al., 1993; Greiner et al., 2008; Saller et al., 2012). Interestingly, ovarian granulosa cells in the follicle express NE as well as all the necessary machinery to transport and release, synthesize, and breakdown NE (Greiner et al., 2008; Saller et al., 2012). Additionally, follicular fluid has been reported to contain levels of NE at least comparable to those of serum in patients (Itoh et al., 2000). Thus, while it has not been empirically shown in the context of HGSOC, it is possible that SPARC remodels the extracellular matrix surrounding the growing ovarian follicle, allowing the NE stored in the granulosa cells to be released from the ovary. We have also previously identified testosterone, canonically stored in ovarian follicles, as being released from the ovary in the presence of tumorigenic FTE, lending further support to this hypothesis (Colina et al., 2021). Once released from the ovary, NE activates adrenergic receptors in fallopian tube derived tumor cells, encouraging invasion into and adhesion to the ovary (Zink et al., 2018). While our current working model for this hypothesis is captured in Figure 6, future experiments could investigate how SPARC may be structurally altering ovarian follicles or structures unique to the human ovary, such as the tunica albuginea, to support our current hypothesis by analyzing granulosa structure before and after exposure to SPARC in co-culture.

Future work could include SPARC knockdown studies with SKOV3 to continue the cross-species comparison of SPARC induced effects on ovarian NE release. Future experiments will also continue to investigate other proteins that may be partially responsible for inducing ovarian NE release. For instance, we intend to investigate the other proteins identified as being in common with MOE PTEN^shRNA^ and SKOV3, but not with MOE SCR^shRNA^, to determine if these factors induce ovarian release of NE individually and increase induction when combined with SPARC. While these future experiments may shed further light on the interaction between FTE and the ovary, the current work discussed here has demonstrated that SPARC secreted from tumorigenic FTE induces ovarian NE release. This discovery presents an opportunity to interrupt the deleterious effects of NE on this system by creating a new point of intervention in the NE release pathway. This intervention can in turn provide a way to disrupt the microenvironmental events contributing to primary metastasis of tumorigenic FTE to the ovary.

## Data Availability

The datasets presented in this study can be found in online repositories. The names of the repository/repositories and accession number(s) can be found below: https://massive.ucsd.edu/ProteoSAFe/dataset.jsp?task=1e835a8628bc414fbcfe32361bc5b6a5, MSV000089866.
