# Supplementary material for: Fallopian tube secreted protein affects ovarian metabolites in high grade serous ovarian cancer
Source: Front Cell Dev Biol. 2022 Nov 7;10:1042734. doi: 10.3389/fcell.2022.1042734 (PMC9676663; doi:10.3389/fcell.2022.1042734)
Supplement: Supplementary file 1 [file DataSheet1.pdf]

Supplementary Material

1 Supplementary Figures and Tables

1.1 Supplementary Figures

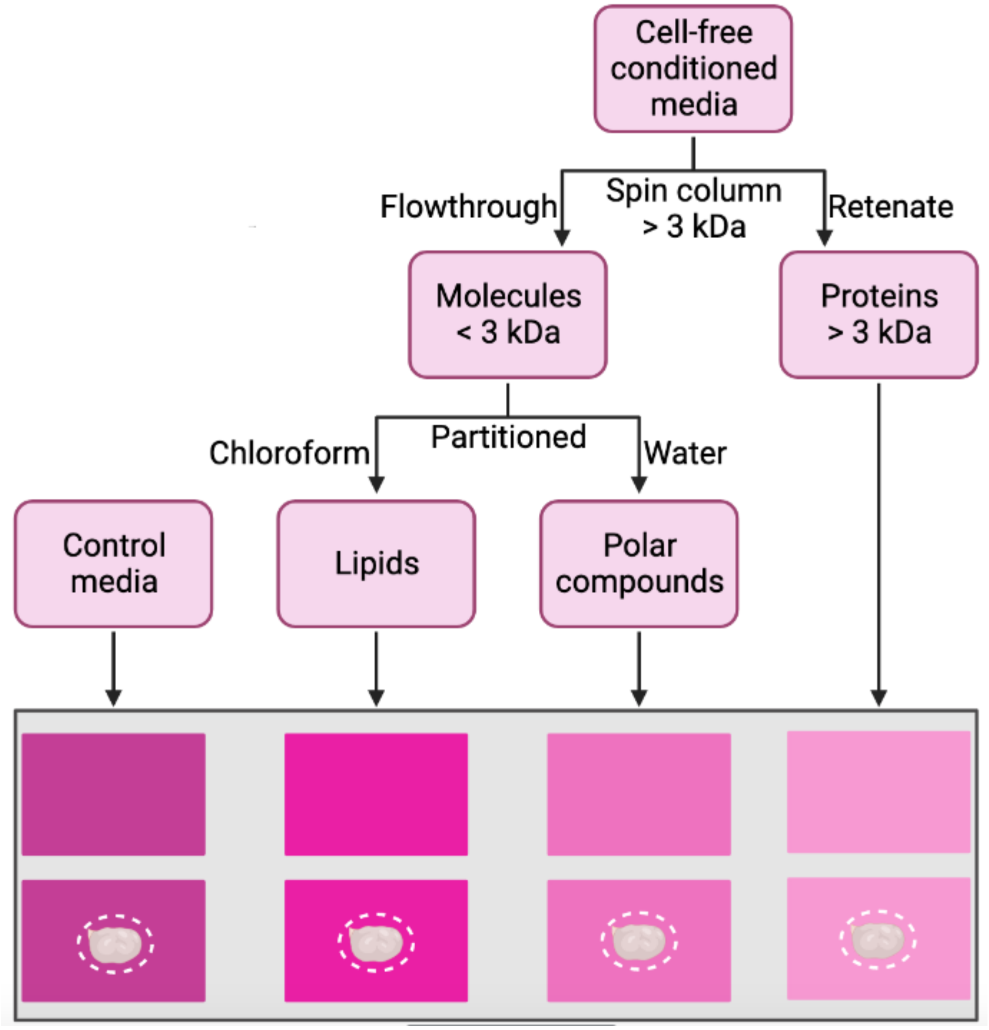

Supplementary Figure 1. Conditioned media from cells was collected and fractionated into three representative biomolecular classes: lipids, polar small molecules, and proteins. Conditioned media aspirated from a T-75 flask was concentrated and filtered through a 3 kDa spin column; the trapped material (> 3 kDa) represents the protein fraction. Control media was not conditioned with cell culture as a negative control.

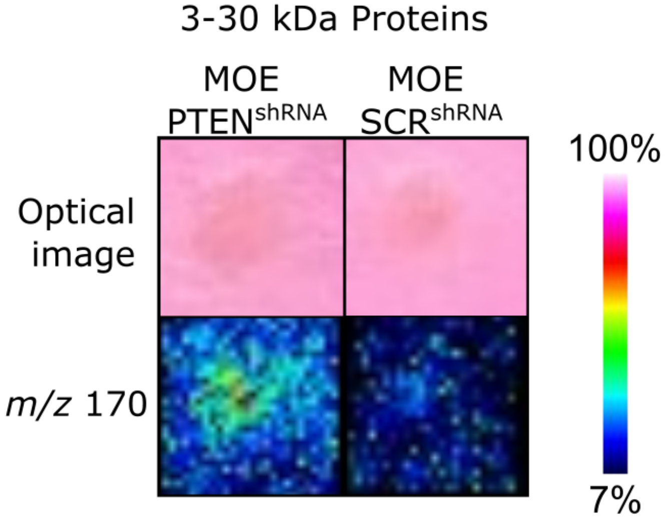

Supplementary Figure 2. Co-culture of 3-30 kDa proteins from each MOE PTEN^{shRNA} and MOE SCR^{shRNA} indicates that the MOE PTEN^{shRNA} conditioned media induce a significant release of NE from the ovarian tissue, and the MOE SCR^{shRNA} conditioned media elicits a weaker signal.

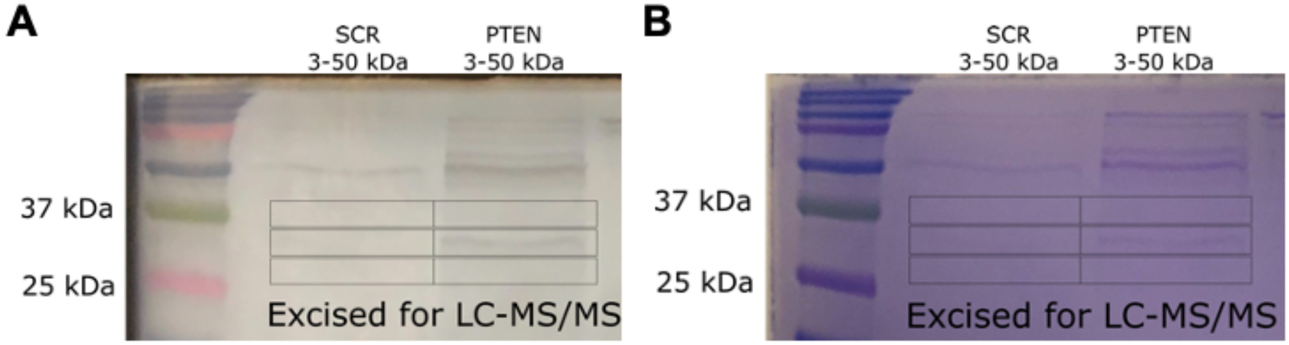

Supplementary Figure 3. SDS-PAGE gel electrophoresis stained with **A)** a Zinc Stain kit and **B)** a Rapid Coomassie Blue Stain kit to compare MOE PTEN^{shRNA} and MOE SCR^{shRNA} proteins. The serum-free MOE PTEN^{shRNA} protein fraction 3-50 kDa exclusively generated a prominent band just below the 37 kDa ladder marker.

1.2 Supplementary Tables

Supplementary Table 1. All proteins from MOE PTEN^{shRNA} and MOE SCR^{shRNA} 3-50 kDa proteomics analysis. H: *Homo sapiens*, M: *Mus musculus*, B: *Bos taurus*.

**MOE PTEN^{shRNA} Only (18)**	**Both (21)**	**MOE SCR^{shRNA} Only (27)**
60S acidic ribosomal protein P2 (M)	(Bos taurus) 54 kDa protein (B)	Alpha-1-antiproteinase (B)
Actin, gamma-enteric smooth muscle (M)	Actin, cytoplasmic 1 (M)	Complement C3 (M)

Alcohol dehydrogenase class 4 mu/sigma chain (M)	Alpha-1-acid glycoprotein (B)	Complement C4 (Fragments) (B)
Beta-2-glycoprotein 1 (B)	Alpha-1B-glycoprotein (B)	Hemoglobin subunit alpha (H)
CCN family member 2 (M)	Alpha-2-HS-glycoprotein (B)	Hemoglobin subunit alpha (M)
Clusterin (M)	Alpha-fetoprotein (B)	Hemoglobin subunit beta (B)
Complement C3 (B)	Apolipoprotein A-I (B)	Histone H3.3 (M)
Cornifin-A (M)	Basal cell adhesion molecule (M)	Keratin, type I cytoskeletal 14 (H)
Cystatin-C (M)	Beta-2-microglobulin (M)	Keratin, type I cytoskeletal 18 (M)
Galectin-1 (M)	Complement factor B (Fragment) (B)	Keratin, type I cytoskeletal 9 (H)
Heat shock protein HSP 90-beta (M)	Fetuin-B (B)	Keratin, type II cytoskeletal 1 (H)
Hemoglobin subunit alpha (B)	Fibrinogen alpha chain (B)	Keratin, type II cytoskeletal 2 epidermal (H)
Hemopexin (B)	Fibronectin (M)	Keratin, type II cytoskeletal 5 (H)
Insulin-like growth factor-binding protein 7 (M)	Glyceraldehyde-3-phosphate dehydrogenase (M)	Keratin, type II cytoskeletal 7 (M)
Malate dehydrogenase, mitochondrial (M)	Histone H2B type 1-B (M)	Metallothionein-1A (B)
Peptidyl-prolyl cis-trans isomerase A (M)	Histone H4 (M)	Metallothionein-2 (M)
Serum amyloid A-3 protein (M)	Osteopontin (M)	Protein AMBP (B)
SPARC (M)	Progranulin (M)	Serotransferrin (H)
	Prosaposin (M)	Syndecan-4 (M)
	Prothrombin (B)	Thrombospondin-1 (Fragment) (B)
	Serotransferrin (B)	Vitamin D-binding protein (B)
	Serum albumin (B)	
	Serum albumin (H)	

	Serum albumin (M)	
	Trypsin OS=Sus scrofa	
	Ubiquitin-60S ribosomal protein L40 (M)	
	Vimentin (M)	

Supplementary Table 2. All proteins from MOE PTEN^{shRNA} and SKOV3 3-50 kDa proteomics analysis. H: *Homo sapiens*, M: *Mus musculus*, B: *Bos taurus*.

SKOV3 Only (37)	Both (14)	MOE PTEN^{shRNA} Only (29)
(Bos taurus) similar to afamin (B)	(Bos taurus) 54 kDa protein (B)	60S acidic ribosomal protein P2 (M)
Alpha-1-antiproteinase (B)	Actin, cytoplasmic 1 (M)	Actin, gamma-enteric smooth muscle (M)
Alpha-enolase (H)	Alpha-1-acid glycoprotein (B)	Alcohol dehydrogenase class 4 mu/sigma chain (M)
Alpha-S1-casein (B)	Alpha-1B-glycoprotein (B)	Apolipoprotein A-I (B)
Alpha-S2-casein (B)	Alpha-fetoprotein (B)	Basal cell adhesion molecule (M)
Annexin A5 (H)	Fetuin-B (B)	Beta-2-glycoprotein 1 (B)
Antithrombin-III (B)	Galectin-1 (M)	Beta-2-microglobulin (M)
Beta-lactoglobulin (B)	Glyceraldehyde-3-phosphate dehydrogenase (M)	CCN family member 2 (M)
Caspase-14 (H)	Hemopexin (B)	Clusterin (M)
Dermcidin (H)	Serotransferrin (B)	Complement C3 (B)
Desmoglein-1 (H)	Serum albumin (B)	Complement factor B (Fragment) (B)
Desmoplakin (H)	SPARC (M)	Complement factor B (Fragment) (B)
Extracellular glycoprotein lacritin (H)	Trypsin OS=Sus scrofa	Cornifin-A (M)
Fatty acid-binding protein 5 (H)	Ubiquitin-60S ribosomal protein L40 (M)	Cystatin-C (M)

Immunoglobulin heavy constant alpha 1 (H)		Fibrinogen alpha chain (B)
Isoform 2 of Triosephosphate isomerase (H)		Fibronectin (M)
Junction plakoglobin (H)		Heat shock protein HSP 90-beta (M)
Kappa-casein (B)		Hemoglobin subunit alpha (B)
Keratin, type I cytoskeletal 10 (H)		Histone H2B type 1-B (M)
Keratin, type I cytoskeletal 14 (H)		Histone H4 (M)
Keratin, type I cytoskeletal 16 (H)		Insulin-like growth factor-binding protein 7 (M)
Keratin, type I cytoskeletal 9 (H)		Malate dehydrogenase, mitochondrial (M)
Keratin, type II cytoskeletal 1 (H)		Osteopontin (M)
